# Maximum likelihood estimation of reviewers' acumen in central review setting: categorical data

**DOI:** 10.1186/1742-4682-8-3

**Published:** 2011-03-25

**Authors:** Wei Zhao, James M Boyett, Mehmet Kocak, David W Ellison, Yanan Wu

**Affiliations:** 1 MedImmune LLC., Gaithersburg, MD, 20878, USA; 2 Department of Biostatistics, St. Jude Children's Research Hospital, Memphis, TN, 38105, USA; 3 Department of Pathology, St. Jude Children's Research Hospital, Memphis, TN, 38105, USA; 4 Department of Mathematical Sciences, University of Memphis, Memphis, TN, 38152, USA

## Abstract

Successfully evaluating pathologists' acumen could be very useful in improving the concordance of their calls on histopathologic variables. We are proposing a new method to estimate the reviewers' acumen based on their histopathologic calls. The previously proposed method includes redundant parameters that are not identifiable and results are incorrect. The new method is more parsimonious and through extensive simulation studies, we show that the new method relies less on the initial values and converges to the true parameters. The result of the anesthetist data set by the new method is more convincing.

## 1. Introduction

Histopathologic diagnosis and the subclassification of tumors into grades of malignancy are critical to the care of cancer patients, serving as a basis for both prognosis and therapy. Such diagnostic schemes evolve, and this process often involves reproducibility studies to ensure accuracy and clinical relevance. However, studies of existing or novel histopathologic grading schemes often reveal diagnostic variance among pathologists[1-4].

The process of histopathologic evaluation is necessarily subjective; even "objective" assessments as part of the histologic work-up of a tumor, such as the mitotic index, are semi-quantitative at best. While this subjectivity underlies discrepancies between pathologists when several evaluate a series of tumors together, a pathologist's experience and skill with different tumor types, especially uncommon tumors such as some brain tumors, will influence his or her performance in this setting. This factor, pathologist "acumen," could be especially influential when new grading schemes are proposed for uncommon tumors. A corollary of this influence is that discussion among a group of pathologists with different levels of experience or acumen about how best to use histopathologic variables in a new tumor-grading scheme might be expected to improve the concordance of their calls. Although estimating inter- and intra-reviewer agreement is important[5-8], in this paper, we are more interested in evaluating the performance of individual reviewers[9,10].

A reviewer's performance can be represented by a matrix , the probability that a reviewer, *k*, records values *l *given *j *is the true category. When the grading category is binary variable,  and  represent the sensitivity or specificity of reviewer *k*, and  and  are the corresponding false-positive or false-negative error rates. When the grading categories are more than two, , *j *≠ *l *are called individual error rates for the *k*^th ^reviewer[9] and(1)

 is defined as the reviewer's acumen because we are more interested in , *j *= 1,...*J *than those error rates. Dawid and Skene[9] proposed a method based on the EM algorithm to estimate . We find that their method has serious drawbacks and may give suspicious results. In particular, their method is over parameterized and doesn't converge to correct parameters for some initial values. We propose a modification to their method, which is also based on the EM algorithm. In the next section, we first derive the incomplete-data likelihood function and then show the EM algorithm solving procedures. We use multiple simulation studies in Section 3 to demonstrate that the new method converges to the correct parameters and relies less on the initial values. Finally, we revisit the anesthetist data used by Dawid and Skene and present a new example of a pathology review data from the Children's Cancer Group (CCG)-945 study[11].

## 2. Model Reviewer's Acumen

Let *X_i _*= (*X_i1_, X_i2_,..., X_iK_*), *i*= 1,2,...,*N*, be the vector of pathologic grades by *K *reviewers for the *i*^th ^sample, in which *X_ik _*is the category assigned by the *k*^th ^reviewer. *X_ik _*is a categorical variable and takes values between 1 and *J*. Let *Y_i _*be the true unknown category, following Bayes' rule the likelihood that the *k*^th ^reviewer classifies the *i*^th ^sample to the *l*^th ^category is written as(2)

where *γ_ij _*= *p*(*Y_i _*= *j*), is the probability that the *i*^th ^sample is truly in category *j *and  is the number of times that a reviewer *k *assigns the sample to category *l*. For most studies,  is either 1 or 0, but it can take values greater than 1 if samples are reviewed multiple times. Assuming that the reviewers work independently, the incomplete-data likelihood function for *K *reviewers is written as(3)

Dawid and Skene used two latent variables to model true category probabilities, a sample specific probability *γ_ij _*(*T_ij _*in the original paper) and population probability *p_j_*, which is the proportion of the *j^th ^*category in the population. Since the estimation of *p_j _*can be expressed as a function of , *p_j _*are redundant and not identifiable. Because of this, the modified model doesn't include *p_j _*in the likelihood function and instead, *p_j _*are expressed as a function of *γ_ij_*.

The overall log-likelihood function is written as(4)

where  and Θ = {*γ_ij_*}. Ω are reviewer specific parameters and Θ are sample specific parameters. In total, there are *K *× *J *× (*J *- 1) + *N *parameters in the model. It is worth noting that the true category probability, *γ_ij_*, is a latent variable and will be estimated in the E step of the EM algorithm.

## 3. Simplex Based EM Algorithm

The method proposed by Dawid and Skene has a closed form solution for , which is derived from the complete data likelihood function. But, their method is overly parameterized, and the convergence relies heavily on the goodness of initial values. It is easy to see that the estimator of  depends solely on its initial values when the estimators of  (equation 2.3 in the original paper) and  (equation 2.4) are put into equation 2.5 in their paper.

The incomplete data likelihood function, equation 4, is a mixture of multinomial probabilities, in which the mixture probabilities, , are unknown. Although solving the incomplete-data likelihood function directly is intractable, one can solve it iteratively using the EM algorithm. The EM algorithm has been widely used to solve mixture models[12], especially those Gaussian mixture models in genetic mapping studies[13]. The same procedures apply here as well. In E step, we estimate the latent variable, , by averaging the posterior probability of the true category over all reviewers. In M step, we use simplex method to search for  that maximize equation 4.

Details of the procedures are as follows:

1. E step: Estimate the  using the posterior probability(5)

where  is from the previous iteration and is considered as a prior probability.

2. M step: Plug  into equation 4 and use the simplex method to search for the  that maximizes the incomplete-data likelihood function,(6)

3. Repeat the E step and M step until convergence.

The simplex algorithm, originally proposed by Nelder and Mead[14], provides an efficient way to estimate parameters, especially when the parameter space is large[13]. It is a direct-search method for nonlinear unconstrained optimization. It attempts to minimize a scalar-valued nonlinear function using only function values, without any derivative information (explicit or implicit). The simplex algorithm uses linear adjustment of the parameters until some convergence criterion is met. The term "simplex" arises because the feasible solutions for the parameters may be represented by a polytope figure called a simplex. The simplex is a line in one dimension, a triangle in two dimensions, and a tetrahedron in three dimensions. Since no division is required in the calculation, the "divided by zero" runtime error is avoided.

## 4. Simulation Study

We design 4 simulation experiments with different sets of reviewers' acumen to test the performance of the proposed method. Each simulation assumes 100 samples, 6 reviewers, and 4 possible grading categories. The first 30 samples are known to be in category 4, the next 30 in category 3, 20 in category 2, and the rest 20 in category 1. In each simulation, we specify  and simulate grading categories according to these probabilities:(7)

Since we are more interested in , only their true and estimated probabilities are given in Tables 1, 2, 3, and 4. The first simulation is the scenario in which all reviewers have good acumen in all categories. Most of them have an 80% chance of making a correct assignment, and only two reviewers in two different categories have a 70% chance. The second simulation assumes that all reviewers have weak acumen in all categories, with only a 50% chance of making correct assignments. The third simulation assumes different reviewers have different acumen in different categories, ranging from 50% to 90%. The last simulation assumes an extreme case, in which 3 reviewers have excellent acumen, a 90% chance, and the other 3 reviewers have weak acumen, only a 50% chance. The estimated values of  shown in Tables 1, 2, 3, and 4 are the average over 1000 repeats, and the numbers in the parentheses are the corresponding square root of mean square errors (RMSE).

**Table 1:** MLE for the first simulation, in which all reviewers had good acumen

	R1	R2	R3	R4	R5	R6
	0.8	0.8	0.8	0.8	0.8	0.8
	0.8	0.8	0.7	0.8	0.8	0.8
	0.8	0.8	0.8	0.8	0.8	0.7
	0.8	0.8	0.8	0.8	0.8	0.8

	0.78 (0.09)	0.78 (0.09)	0.78 (0.1)	0.78 (0.09)	0.78 (0.09)	0.78 (0.1)
	0.78 (0.09)	0.78 (0.09)	0.69 (0.11)	0.78 (0.09)	0.78 (0.09)	0.78 (0.1)
	0.8 (0.08)	0.79 (0.07)	0.8 (0.09)	0.8 (0.08)	0.8 (0.07)	0.7 (0.1)
	0.8 (0.08)	0.8 (0.08)	0.8 (0.09)	0.8 (0.08)	0.8 (0.08)	0.81 (0.09)

**Table 2:** MLE for the second simulation, in which all reviewers had weak acumen

	R1	R2	R3	R4	R5	R6
	0.5	0.5	0.5	0.5	0.5	0.5
	0.5	0.5	0.5	0.5	0.5	0.5
	0.5	0.5	0.5	0.5	0.5	0.5
	0.5	0.5	0.5	0.5	0.5	0.5

	0.45 (0.16)	0.46 (0.15)	0.48 (0.15)	0.47 (0.16)	0.49 (0.15)	0.49 (0.15)
	0.45 (0.16)	0.46 (0.15)	0.47 (0.16)	0.48 (0.16)	0.48 (0.15)	0.5 (0.15)
	0.51 (0.15)	0.52 (0.15)	0.52 (0.15)	0.53 (0.14)	0.54 (0.14)	0.54 (0.14)
	0.54 (0.16)	0.54 (0.16)	0.54 (0.16)	0.53 (0.15)	0.53 (0.15)	0.53 (0.15)

**Table 3:** MLE for the third simulation, in which reviewers had mixed acumen

	R1	R2	R3	R4	R5	R6
	0.5	0.9	0.9	0.7	0.9	0.9
	0.7	0.9	0.9	0.9	0.5	0.9
	0.8	0.7	0.6	0.9	0.9	0.9
	0.8	0.9	0.6	0.9	0.7	0.9

	0.5 (0.16)	0.88 (0.11)	0.88 (0.16)	0.69 (0.14)	0.88 (0.18)	0.87 (0.07)
	0.7 (0.16)	0.87 (0.11)	0.88 (0.17)	0.87 (0.11)	0.5 (0.2)	0.86 (0.08)
	0.8 (0.14)	0.7 (0.12)	0.6 (0.17)	0.89 (0.11)	0.9 (0.17)	0.88 (0.06)
	0.81 (0.14)	0.91 (0.1)	0.6 (0.18)	0.9 (0.1)	0.7 (0.19)	0.9 (0.06)

**Table 4:** MLE for the fourth simulation, in which some reviewers had good acumen and some had weak acumen

	R1	R2	R3	R4	R5	R6
	0.5	0.5	0.5	0.9	0.9	0.9
	0.5	0.5	0.5	0.9	0.9	0.9
	0.5	0.5	0.5	0.9	0.9	0.9
	0.5	0.5	0.5	0.9	0.9	0.9

	0.5 (0.11)	0.5 (0.12)	0.5 (0.12)	0.86 (0.08)	0.86 (0.08)	0.86 (0.08)
	0.5 (0.12)	0.5 (0.12)	0.5 (0.12)	0.86 (0.08)	0.86 (0.08)	0.86 (0.08)
	0.5 (0.09)	0.51 (0.1)	0.51 (0.09)	0.89 (0.06)	0.88 (0.07)	0.88 (0.06)
	0.51 (0.09)	0.51 (0.09)	0.51 (0.1)	0.91 (0.06)	0.9 (0.06)	0.9 (0.06)

The estimated values for  in all 4 simulation studies converge to true parameter values. The probabilities for categories 3 and 4 are closer to the true values, and the RMSEs are smaller. This is what is expected because categories 3 and 4 have 10 more samples than categories 1 and 2. In general, the RMSE is higher for small probabilities and smaller for large probabilities. In addition, the values for , *l *≠ *j *converge to the true values as well(data not shown).

To show that our method is less dependent on initial values, we used non-informative initial values in our simulation studies, i.e.  and(8)

In Dawid and Skene method,  is a saddle point, at which the method converges to itself if used as initial values. However, these initial set of values work well in our method. We define that the computation reaches convergence when the log likelihood function between two iterations is less than 10^-3^. Although more stringent threshold can be used, we find that 10^-3 ^is generally sufficient to guarantee convergence.

## 5. Examples

### 5.1 Revisit the Anesthetist data

This data set was used by Dawid and Skene for a demonstration of their method. Briefly, the data came from five anesthetists who classified each patient on a scale of 1 to 4. Anesthetist 1 assessed the patients three times, but we assume that the assessments were independent, as did by the previous authors. Table 4 in their paper gives the estimated probabilities *γ_ij _*for each patient. Most estimates in the table are either 1 or 0, which is very unlikely given the level of disagreement between reviewers in the study.

In the data, observer 1 assigned patient #36 to category 3 twice and category 4 once, observers 2 and 4 assigned the same patient to category 4, and both observers 3 and 5 assigned him to category 3. It was estimated that the patient had 100% probability of being in category 4, . After closely examining the data, we found that category 4 was actually the category to which all observers assigned patients least frequently, and patient #11 was the only one all observers agreed on as being in category 4 and there was no extra data to establish acumen in this category for any reviewers. Because of this observation, their estimate of patient category probability is unrealistic and suspicious. For patient #3, reviewer 1 gave category 1 twice and category 2 once; reviewers 2, 4, 5 gave category 2 and reviewer 3 gave category 1. The patient was estimated 100% in category 2. Results for patients 2, 10, and 14 are also suspicious.

We reanalyzed the anesthetic data using our method. The acumen estimates are given in Table 5 and the estimated category assignment for each patient is given in Table 6. For patient #36, we estimated that there was 73% chance that the patient was in category 3 and a 27% chance he was in category 4. Patient #3 was estimated to have 50% chance of being in either category 1 or 2. Our estimates are more realistic.

**Table 5:** MLE of the observers' acumen (individual error rate) from the anesthetic data

			Observer 1		
	Observed Response	1	2	3	4
True Response	1	0.87	0.13	0	0
	2	0.03	0.88	0.09	0
	3	0	0.03	0.9	0.07
	4	0.01	0.05	0.07	0.87
					
			**Observer 2**		
	**Observed Response**	**1**	**2**	**3**	**4**

True Response	1	0.79	0.21	0	0
	2	0.05	0.65	0.3	0
	3	0	0	0.61	0.39
	4	0.01	0.07	0.04	0.89
					
			**Observer 3**		
	**Observed Response**	**1**	**2**	**3**	**4**

True Response	1	0.92	0.07	0.01	0
	2	0.04	0.83	0.13	0
	3	0	0.22	0.39	0.39
	4	0.1	0.08	0	0.81
					
			**Observer 4**		
	**Observed Response**	**1**	**2**	**3**	**4**

True Response	1	0.88	0.12	0	0
	2	0.05	0.76	0.14	0.06
	3	0	0	0.8	0.2
	4	0.03	0.26	0.1	0.62
					
			**Observer 5**		
	**Observed Response**	**1**	**2**	**3**	**4**

True Response	1	0.92	0.07	0.02	0
	2	0.19	0.63	0.18	0
	3	0	0.27	0.55	0.18
	4	0	0	0.01	0.98

**Table 6:** Estimated category probability for each patient for the anesthetist data

		Category					Category		
			
Patient	1	2	3	4	Patient	1	2	3	4
1	1	0	0	0	24	0.14	0.86	0	0
2	0	0	0.95	0.05	25	1	0	0	0
3	0.5	0.5	0	0	26	1	0	0	0
4	0.24	0.76	0	0	27	0	0.93	0.07	0
5	0	1	0	0	28	1	0	0	0
6	0	1	0	0	29	1	0	0	0
7	0.68	0.32	0	0	30	0.82	0.18	0	0
8	0	0	1	0	31	1	0	0	0
9	0	1	0	0	32	0	0	1	0
10	0	0.85	0.15	0	33	1	0	0	0
11	0	0	0	1	34	0	1	0	0
12	0	0.65	0.35	0	35	0	0.93	0.07	0
13	1	0	0	0	36	0	0	0.73	0.27
14	0.11	0.89	0	0	37	0.14	0.85	0.02	0
15	0.99	0.01	0	0	38	0	0.51	0.49	0
16	1	0	0	0	39	0	0	1	0
17	1	0	0	0	40	1	0	0	0
18	1	0	0	0	41	1	0	0	0
19	0	1	0	0	42	0.89	0.11	0	0
20	0.1	0.9	0	0	43	0	0.93	0.07	0
21	0	1	0	0	44	0.99	0.01	0	0
22	0	1	0	0	45	0	1	0	0
23	0	1	0	0					

### 5.2 Empirical Study: CCG-945

In the CCG-945 study[11], sections of study tumors were centrally reviewed, initially by a study review neuropathologist and subsequently by 5 neuropathologists, including the review pathologist. The review neuropathologist, who was masked to institutional diagnoses and his original review diagnoses, provided revised review diagnoses based on the revised WHO criteria[15], and that review was used to establish the consensus diagnosis with the independent, concurrent reviews of 4 other experienced neuropathologists who were masked to outcome. There were 172 randomized patients reviewed in CCG-945. Five central reviewers classified tumors into 4 grading categories: 1 = anaplastic astrocytoma (AA); 2 = glioblastoma multiforme (GBM); 3 = other high-grade glioma; and 4 = not high-grade glioma (Pollack et al., 2003) [11]. Category 3 is rather heterogeneous and contains all other high-grade glioma other than AA and GBM. It was the least frequently used category by all reviewers. The estimated acumen for each reviewer is shown in Table 7.

**Table 7:** MLE of the reviewers' acumen for the CCG-945 data

			Reviewer 1		
	Observed Response	1	2	3	4
True Response	1	0.78	0.12	0.09	0.00
	2	0.15	0.85	0.00	0.00
	3	0.49	0.15	0.30	0.07
	4	0.13	0.02	0.09	0.76
					
			**Reviewer 2**		
	**Observed Response**	**1**	**2**	**3**	**4**

True Response	1	1.00	0.00	0.00	0.00
	2	0.42	0.52	0.03	0.03
	3	0.32	0.15	0.32	0.20
	4	0.00	0.00	0.07	0.93
					
			**Reviewer 3**		
	**Observed Response**	**1**	**2**	**3**	**4**

True Response	1	0.79	0.15	0.00	0.06
	2	0.08	0.88	0.00	0.04
	3	0.22	0.15	0.38	0.26
	4	0.32	0.04	0.05	0.60
					
			**Reviewer 4**		
	**Observed Response**	**1**	**2**	**3**	**4**

True Response	1	0.62	0.21	0.01	0.15
	2	0.14	0.76	0.06	0.03
	3	0.00	0.29	0.58	0.13
	4	0.02	0.00	0.06	0.93
					
			**Reviewer 5**		
	**Observed Response**	**1**	**2**	**3**	**4**

True Response	1	0.82	0.06	0.06	0.07
	2	0.30	0.68	0.00	0.02
	3	0.51	0.13	0.36	0.00
	4	0.25	0.04	0.13	0.58

It is interesting to see that reviewers have different level of acumen to differentiate AA from GBM based on the revised WHO criteria. If we assume 80% sensitivity (or specificity) is an indicator of good acumen, reviewers 1 and 3 are very experienced in grading AA and GBM, and reviewer 2 clearly needs some improvement. None of the reviewers did well in grading category 3, i.e. other high-grade gliomas. This is somewhat expected because it is the least frequent and most heterogeneous category. When the true category is 4, reviewers 1, 3, and 5 all assigned a noticeable proportion to category 1. The reason may be that some low-grade gliomas in category 4 are difficult to differentiate from AA according to WHO criteria.

## 6. Conclusion

The method developed by Dawid and Skene was based on the EM algorithm. It starts with a complete data likelihood function, and then  has a closed form solution. Their method only requires initial values for , which are reasonable, non-informative initial values, but they are saddle points of the complete data likelihood function. The method does not converge from these initial values at all. Alternative initial values (equation 9) calculated from the data were proposed to address this issue(9)

However, when their method converges, it may converge to suspicious results, as was shown in their example.

Our method is less dependent on initial values and converges to similar values from any reasonable initial values. Because our method starts with the incomplete data likelihood, there is no closed form solution for , and solving equation 4 directly is intractable. We adopted the EM algorithm, which is widely used in solving Gaussian mixture models, for this formidable task. In the M step, we used the simplex method to search for parameters that maximize the incomplete data likelihood function.

In cases when a reviewer is uncertain about a particular sample, the same sample can be recorded multiple times to different categories. No modification to the model is necessary. Using simulation studies, we have shown that our method performs well at a variety of scenarios with fairly small sample sizes. Our model has *K *× *J *× (*J *- 1) + *N *parameters, *J-1 *fewer than Dawid and Skene's model. Because the model is highly parameterized, it would be naive to expect any of the theoretical large sample optimality properties to hold[9]. This work focuses entirely on estimating reviewers' acumen, and no hypothesis testing is discussed. We believe that the issue of hypothesis testing can be addressed using a likelihood ratio test[16] and bootstrap method[17]. The reliability of the parameter estimation can be assessed using bootstrap method techniques as well, but it is not the focus of this work. The R program used for the simulation studies and for analyzing the anesthetic data is available upon request.

## Competing interests

The authors declare that they have no competing interests.

## Authors' contributions

WZ drafted the manuscript, developed the statistical method, and performed simulation and data analysis. JB provided the data and provided substantial contribution to the conception of the method. MK provided important comment to improve the method. DWE wrote part of the introduction and provided insight from a pathologist's viewpoint. YW helped to test the method and edit the manuscript. All authors read and approved the final manuscript.

## References

[B1] Stenkvist B Bengtsson E Eriksson O Jarkrans T Nordin B Westman-Naeser S Histopathological systems of breast cancer classification: reproducibility and clinical significance J Clin Pathol 1983 36 392 398 10.1136/jcp.36.4.392 6833508 PMC498233

[B2] Tihan T Zhou T Holmes E Burger PC Ozuysal S Rushing EJ The prognostic value of histological grading of posterior fossa ependymomas in children: a Children's Oncology Group study and a review of prognostic factors Mod Pathol 2008 21 165 177 18084249 10.1038/modpathol.3800999

[B3] Longacre Teri A Ennis Marguerite Quenneville Louise A Bane Anita L Bleiweiss Ira J Carter Beverley A Catelano Edison Hendrickson Michael R Hibshoosh Hanina Layfield Lester J Memeo Lorenzo Wu Hong O'Malley Frances P Interobserver agreement and reproducibility in classification of invasive breast carcinoma: an NCI breast cancer family registry study Mod Pathol 2006 19 195 207 10.1038/modpathol.3800496 16341153

[B4] Izadi-Mood Narges Yarmohammadi Maryam Ahmadi Seyed Ali Irvanloo Guity Haeri Hayedeh Meysamie Ali Pasha Khaniki Mahmood Reproducibility determination of WHO classification of endometrial hyperplasia/well differentiated adenocarcinoma and comparison with computerized morphometric data in curettage specimens in Iran Diagnostic Pathology 2009 4 10 10.1186/1746-1596-4-10 19317919 PMC2681452

[B5] Cohen Jacob A coefficient of agreement for nominal scales Educational and Psychological Measurement 1960 20 1 37 46 10.1177/001316446002000104

[B6] Fleiss JL Statistical methods for rates and proportions 1981 New York: John Wiley

[B7] Landis JR Koch GG The measurement of observer agreement for categorical data Biometrics 1977 33 159 174 10.2307/2529310 843571

[B8] Barnhart HX Williamson JM Modeling concordance correlation via GEE to evaluate reproducibility Biometrics 2001 57 931 940 10.1111/j.0006-341X.2001.00931.x 11550947

[B9] Dawid P Skene AM Maximum likelihood estimation of observer rates using the EM algorithm Journal of the Royal Statistical Society. Series C (Applied Statistics) 1979 28 1 20 28

[B10] Hui Siu L Zhou Xiao H Evaluation of diagnostic tests without gold standards Statistical Methods in Medical Research 1998 7 354 370 10.1191/096228098671192352 9871952

[B11] Pollack Ian F Boyett James M Yates Allan J Burger Peter C Gilles Floyd H Davis Richard L Finlay Jonathan L for the Children's Cancer Group The influence of central review on outcome associations in childhood malignant gliomas: Results from the CCG-945 experience Neuro-Oncology 2003 5 197 207 10.1215/S1152851703000097 12816726 PMC1920685

[B12] Hastie Trevor Tibshirani Robert Friedman Jerome The EM algorithm The Elements of Statistical Learning 2001 New York: Springer

[B13] Zhao W Wu RL Ma C-X Casella G A fast algorithm for functional mapping of complex traits Genetics 2004 167 2133 2137 10.1534/genetics.103.024844 15342547 PMC1471016

[B14] Nelder JA Mead R A simplex method for function minimization Comput J 1965 7 308 313

[B15] Kleihues P Burger PC Scheithauer BW Histological typingof tumours of the central nervous system International Histological Classification of Tumours 1993 21 11 16

[B16] Casella G Berger RL Statistical Inference 2001 Belmont: Duxbury Press

[B17] Efron B Tibshirani RJ An introduction to the bootstrap 1993 Boca Raton:Chapman & Hall/CRC

